# The Remote Cause of Scrofulous Forms of Disease

**Published:** 1863-10

**Authors:** W. Byrd Powell

**Affiliations:** Covington, Ky.


THE
### CHICAGO MEDICAL EXAMINER.

N. S. DAVIS, M.D., Editor.

VOL. IV. OCTOBER, 1863. NO. 10.

### Original Contributions.

### ARTICLE XXV.

### THE REMOTE CAUSE OF SCROFULOUS FORMS OF DISEASE.

By W. BYRD POWELL, M.D., of Covington, Ky.

Mr. Editor:—The very favorable reception of my last contribution, as indicated by the many letters I have received in relation to it, and that too, immediately upon its reception, encourages me to send you another, which has some affinity with the last and, probably, of equal moment.

The ignorance of our profession, of the remote cause of scrofulous forms of disease, has for centuries been denominated the *approbrium medicorum.* But, sir, it can no longer be preferred, because I have discovered it; and that I have, I am just as confident as I am of any other proposition in the profession; and now I submit it to the profession, hoping that it may command the most searching observation. When scrofulous forms of disease are met with, they are, to the extent of my observation, attributed to an hereditary diathesis.

I am entirely certain that not less than ninety-five per centum of our scrofulous forms of disease are entirely independent of any such cause. I grant, however, that the scrofulous diathesis is hereditary, but I am not prepared to admit that it occasionally skips a generation and reappears. I grant, however, that a generation may escape scrofulous forms of disease, but it is because it escapes adequate cause of excitement; but the diathesis continues to be transmitted as long as it exists, while the certain index of its existence is not generally known. A feeble endowment of the motory centre is, I hold, an invariable index of the scrofulous diathesis; and this centre is in the cerebellum, midway between the mesial line and the mastoid processes, respectively.

Within a recent period, comparatively, considerable has been written in relation to the remote cause of scrofulous forms of disease. Dr. CUMIN, in Cyclopædia of Practical Medicine, article Scrofula, says:—"In considering the causes of a disease so deeply rooted in the constitution, as scrofula is universally acknowledged to be, it is necessary to direct our attention to circumstances very remote in the history of its subjects. The foundation of a scrofulous habit is frequently laid during the fœtal state, by the transmission of that peculiar organization of the frame from parents who themselves possess it." The Dr. was right in going far back in the history of scrofulous subjects to seek the cause, but he did not go back sufficiently far,—he only went back far enough to find a proximate cause, but not the remote one,—he did not find the remote cause of the first case that announced the dawn of civilization. The diathesis must have first been produced before it could have become hereditary.

Again, he says, "a cold and variable climate, like those of Great Britain and Holland, is particularly favorable of scrofula; in proof of which, it is sufficient to adduce the great prevalence of the malady in both of these countries." Concede the verity of this statement, and nothing more is gained than that these countries are well adapted to the development of a scrofulous diathesis into a scrofulous disease,—the cause of the diathesis is still undiscovered. Prof. EBERLE, in his Practice, by MCCLELLAND, p. 754, attributes the scrofulous diathesis to, 1. Climate and atmospheric influences; 2. To impure and confined air of cities; 3. To deficient and unwholesome food; and, 4. To various forms of disease. All these may be very efficient in exciting into activity a pre-existing diathesis, but we have had no evidence, that can be regarded as satisfactory, that either or all of them can produce the scrofulous diathesis. Scrofula obtains greatly in Virginia, where the climate is genial and one of the best; and the people have always been distinguished for being good and generous livers. Scrofula also abounds in Massachusetts. The climate, it is true, is cold but steady; and no people are, probably, more comfortable livers, generally. Perhaps, no country furnishes more squalid poverty than Ireland, and yet the Irish are less scrofulous than their English neighbors. Scrofula is by no means common to our wild tribes of Indians, and yet they are very little more protected against atmospherical vicissitudes than are the forest animals, and during much of their time they are stinted in food, and the little they have is of the poorest quality. I have known entire families of Indians, women and children, to live through the winter exclusively on bitter acorns. Scrofula is also almost unknown to our frontier people, and very generally they are but poorly fed, clothed, or sheltered. If the scrofulous diathesis obtained with either them or the Indians, scrofula would equally abound, because they are constantly exposed to the most adequate causes of excitement.

Prof. WOOD, in his Practice, Vol. I., p. 114, has taken a more correct view of this subject than has any one else whom I have consulted. After alluding to the usually assigned causes for the scrofulous diathesis, particularly those above-named, he says, "There are many individuals upon whom all those causes may be made to operate, and so intensely too, as even to produce fatal effects, without giving rise to this particular disease. Indeed, it is probable that the great majority of mankind might perish under these circumstances and give no sign of the disease. There is something more, therefore, than mere debility. There is some inherent peculiarity of the organization, generally derived from the parents, which serves as the basis of the disease. The other causes are, in general, merely exciting. They no doubt induce the disease, when it might otherwise never have been developed; but they are, generally, incapable of producing it, unless in subjects having some innate disposition towards it. In the great majority of fatal cases, the original and essential cause will, probably, be found to be an inherited peculiarity of the organization."

Prof. WOOD has very candidly led us to the inference, that of the cause of the scrofulous diathesis he knows nothing, but entertains the opinion that it is derived from the progenitors. Dr. CUMIN was of the same opinion, with this difference, he thought it was derived from such parents as had themselves the diathesis. Prof. WOOD does not specify any condition of the parents, hence, we are at liberty to infer that it may be derived from such as are normally organic and physiological; and this must be conceded to be the fact before we can reasonably expect to find the remote cause of the first case of scrofula that ever obtained in our race, and this is exceedingly desirable, because that remote cause may still be operating, and, possibly, with more power and frequency than ever before, and that this is the fact I doubt not; and it must be discovered before we can hope to emancipate our race from scrofulous forms of disease.

I am as confident as I am of any physiological truth, that the remote cause of all truly scrofulous forms of disease originates in a physiological incompatibility of progenitors. Dr. CUMIN, in searching for the cause of the scrofulous diathesis, went back to the fœtus; he found a cause, but it was not *the* cause. The cause of the scrofulous diathesis had also a cause, and I have discovered it to be, almost exclusively, civilization. To a very limited extent it is incidental to primitive humanity, in some of its relations, hence, scrofula, to a limited extent, obtains with our primitive people, the Indians. When I discovered it to be a fact, that the most sound and physiological of our respective sexes, in marriage, are frequently so physiologically incompatible as to entail a scrofulous diathesis on their children, the fact, *a priori*, seemed to me to be unreasonable and unphysiological, and, hence, I was greatly perplexed. But having ascertained the fact beyond the possibility of a doubt; I sought its rationale, and have most satisfactorily obtained it.

Ninety-nine per centum of our incompatible marriages are occasioned by the lymphatic and the encephalic temperaments, and a proper understanding of their origin, respectively, most thoroughly illuminates the subject. The lymphatic and the encephalic temperaments obtain only under the influences of civilization, they do not obtain with primitive people. One exception may be cited against me, but it is an error. Some physiologist, name not remembered, represents the Esquimaux people as being lymphatic, and they are primitive. When I saw this statement, I was strongly persuaded that it was an error; and having since obtained five of their crania, I find them to be bilious. They are an obese people; indeed, obesity is an essential condition of animal life in that climate,—a lymphatic person could neither originate nor live there.

I assume wealth to have originated with civilization, because the fact is universally conceded; wealth induces relaxation from toil, and many varieties of personal indulgence, and these enfeeble the physiological energies, and a lymphatic repletion is the consequence. Primitive and frontier people have to be in constant exertion to obtain the means of subsistance and, therefore, not permitted to become lymphatic. About our drinking-saloons and other haunts of idleness, people may be constantly seen who are rapidly becoming lymphatic; and, when this repletion is induced, a lymphatic diathesis becomes entailed. Care, responsibility, and mental activity, generally, are about as exclusively incidental to civilization as wealth is; and, thus, the cerebrum is developed to the neglect of the cerebellum; and, hence, a physiological debility as certainly eventuates as with the lymphatic condition. The constitution thus induced is the encephalic. Any observing physician may witness the constant development of the lymphatic and encephalic temperaments amongst his acquaintances.

The Hollanders are notoriously scrofulous and as notoriously thrifty, and, consequently, the lymphatic and encephalic temperaments are so exceedingly diffused amongst them that I think it very probable that not even one per centum of their marriages is physiological, and, hence, in about the same proportion their people have a scrofulous diathesis; and as their climate is exceedingly favorable to develope the diathesis into scrofulous forms of disease, we can now understand why it is the Hollanders are so scrofulous. The great commercial prosperity of the English people and their climate, are rapidly rendering them as scrofulous as the Hollanders. Indeed, all the prosperous states of Europe are suffering greatly from the scourge of scrofula.

Such has been the extraordinary prosperity of our people for thirty-five years preceding our Civil War that the lymphatic and encephalic temperaments have, in that time, not only been increased but wonderfully disseminated amongst our people. Having been an active observer in this relation for forty years, I know that about which I write. As early as 1830, it was a novelty to see any one who participated in the encephalic temperament, but now it obtains in very nearly a-third of society, particularly in our cities. At the time above mentioned, the number was small who had a lymphatic repletion of the body, but now they are as plenty as blackberries in July; and the result of this great dissemination of the lymphatic encephalic constitutions amongst our people, is, that our physiological marriages are reduced to two-sevenths. Every physician of my age is aware of the fact, that scrofulous forms of disease have astonishingly increased in the preceding thirty years. Idiocy and imbecility are two of the results of incompatible marriage; and before 1830 there was not, I think, in our whole country a single asylum for the care of such children; but now almost every State has such an institution, and some two or more. This is significant. Some aged physicians think that all forms of disease are now less manageable than they were thirty years ago. This, I doubt not, is true, but it has not been because of any essential change in the abstract character of disease, but because the constitution of our people has become more or less depraved by the scrofulous diathesis, and is, therefore, less able than formerly to contend with disease. Physicians are now heard to say, that no skill can cure the children of some people when they become sick. This is true; but whose children are these? I answer, confidently, they are the strumous apologies for children of incompatible progenitors. Some physicians, in Cincinnati, form their prognosis as soon as they see the parents. If they are compatible, the child can be cured; but, if otherwise, the child will die. All physicians may acquire this ability by the study of my Science of Physiological Marriage, as epitomized in the May and June Nos. of the *Examiner*.

Before the dawn of civilization, man's native instincts were sufficient to guide his matrimonial relations,—as they still are with uncivilized people; but the introduction, by civilization, of two constitutional conditions has placed the marriage relation beyond the pale of his instincts. Civilization has made the discovery of many sciences essential to man in his civilized state, and among them is the new science of physiological marriage; and it must be conceded that it is the most essential science ever announced for the consideration of man; and, if he neglect it, the premature extinction of his race is just as certain as it is that the sun rises in the east and will continue to do so, for the natural laws govern both.

Our Civil War is doing much to mitigate the scourge of scrofula, particularly in the Confederate States, but more particularly for the black population; for, before the Rebellion, slave property was depreciating, because of the prevalence of the scrofulous diathesis. As paradoxical as it may seem, it is nevertheless true, that war, pestilence, and famine are the great conservatives of our race, and, when they shall be no more, our race will soon be no more.

I am strongly inclined to believe that every physician in our country, who has had for a few years a tolerable practice, must remember enough of his observations to incline him to think it probable that the cause of the scrofulous diathesis is physiologically incompatible marriage. Consanguine marriages have been thought to be a source of the scrofulous diathesis; but this opinion I am confident is entitled no more consideration than any other prejudice, for as frequently as I have found consanguine parties to be temperamentally compatible, so frequently have I found their children all right and without any distinction in relation consanguinity. When I find married parties to be temperamentally incompatible, I find the children with a scrofulous diathesis.

In concluding this thesis, I beg leave to cite one case as being illustrative of the science of physiological marriage and also of the remote cause of scrofulous forms of disease:—

A year or so since, one of the most able and distinguished theologians known to the Christian church of this or any other country called on me, introduced himself, and said:—"Are you Prof. POWELL?" I am, sir. "I have learned from some friends that you have made a highly interesting discovery in human physiology, and, as I have always been much interested in human physiology, I have called to get you to explain it to me, as my friends could not do it satisfactorily, provided it will be agreeable to you?" It will afford me pleasure, sir, please be seated. I explained to him the character of the physiological incompatibility that frequently happens between the most sound and physiological parties of our respective sexes in marriage, and the consequences. I found him by the by to be an extensively informed physiologist, and much regret that propriety forbids the promulgation of his name.

Upon the conclusion of my explanation, he said, "Suppose you see one of the parties to a marriage and have a description of the other, can you then infer any thing of their children?" I can, sir, if the description be correct. "Then, sir, I will give you a description of my wife." He described her, and said, "what do you suppose her temperament to be?" Bilious lymphatic, with a considerable predominance of the bilious. "Just so, sir, I think you are right; that has always been my opinion." "What is my temperament?" Sanguine bilious encephalic, with a strong predominance of the sanguine and bilious elements. "Now, if you please, your opinion of our children?" I think, sir, you have had a numerous progeny; that they, respectively, lived to adult age with a fair promise of age and usefulness; but, sir, before the age of thirty years they, respectively, died of phthisis. He sprang to his feet, exclaiming "stop, sir! stop, sir! stop!" What, sir. "I wish to inform you that consumption was never an heirloom in my ancestry nor in my wife's, for I have the history of both for many generations in Europe." Having had no information in relation to your ancestors, respectively, I assumed your statement as being true when I gave my opinion of your children; nevertheless, sir, I may have erred, but, if I have, it was because of some misapprehension of your respective constitutions; but, not being conscious of any such misapprehension, I have no modification of my opinion to offer; and, if you are as old as I think you are, your children are all dead, and you know the fact and also the affection of which they died. "What has my age to do with it?" Nothing, sir, more than I have stated,—you have been permitted to know all the facts. "But, Professor, I conceive that there is a very formidable difficulty in the way of your opinion." Name it, sir? "I have been a physiological reader all my life, and have thus learned that when both parties to a marriage have good health and sound constitutions, their children will also; this I understand to be a settled doctrine in physiology. What are you to do with all this authority against your opinion? For my wife and I thoroughly fill the conditions; for a more sound and healthy couple than we are, and always have been, do not live in this or any other country." The authority you have named, sir, is not in my way, and as you have been extensively a physiological reader, you must be aware that I am the only one who has taught that the respective sexes of our species, however sound they may be, are frequently so incompatible, constitutionally, as to entail a scrofulous diathesis on their children. It is to such an incompatibility between you and your wife that I attribute your bereavements. "To the extent of my reading, you are the first and only one who has taught this doctrine, and you are a master of it in all its relations."

"I will give you the facts: my wife brought me just one dozen children, and all of them lived to adult age, and some of them married and had children, but, sir, neither of them lived to the age of thirty years,—all died of phthisis, and I am now a childless old man. But, sir, I confess myself actually unable to comprehend how, on a subject of this kind, you can arrive at such a discriminating precision as you have manifested in relation to my family." To learn that I have investigated this subject sixteen years will, probably, help you. "No, sir, if you had investigated sixteen hundred years the difficulty would not be materially reduced, for it is neither chemistry nor mathematics. I can only regard your decisions as ultimate manifestations of the human mind. That you have made a discovery I cannot doubt, and one too that will astonish all the world. I will not say that it is the greatest discovery ever made, but, sir, it is the most important, because it most directly concerns the perpetuity of our race, which is confessedly the most important interest of humanity. You must prepare yourself for much incredulity, for this discovery will have more to contend with than any other ever had, because the subject has been thought to be the most inscrutable. It seems to me that your discovery of sexual incompatibility reveals another of no less brilliancy, and of which, possibly, you may not have thought. It is this: it reveals the cause of the scrofulous diathesis. As I was confident that such a diathesis was not in either my wife nor myself, I felt confident that I would balk you when you said, my children would die of phthisis, but I was mistaken. I have presented the facts of my family to many eminent physicians in both this country and Europe, but you alone have consistantly explained them; and you have given the only explanation that can be given, I think. The cause of the scrofulous diathesis has engaged the first minds of your profession for more than two thousand years, and that you have discovered it, my own case is as strong proof as can be had."

